# Community-Metabolome Correlations of Gut Microbiota from Child-Turcotte-Pugh of A and B Patients

**DOI:** 10.3389/fmicb.2016.01856

**Published:** 2016-11-16

**Authors:** Xiao Wei, Shan Jiang, Xiangna Zhao, Huan Li, Weishi Lin, Boxing Li, Jing Lu, Yansong Sun, Jing Yuan

**Affiliations:** Institute of Disease Control and Prevention, Academy of Military Medical Sciences, Fengtai DistrictBeijing, China

**Keywords:** metabolome, liver cirrhosis, gut microbiota, SCFAs, amino acid

## Abstract

The gut flora are widely involved in the cometabolism with the host and have evident effects on the metabolic phenotype of host. This study performed a metabolome analysis of the intestinal microbiota specific for liver cirrhosis. The study population included patients with Child-Turcotte-Pugh score of A (AP, *n* = 5) and B (BP, *n* = 5), and control subjects (NM, *n* = 3). Metagenomic DNA from fecal microbiota was extracted followed by metagenomic sequencing through Illumina MiSeq high throughput sequencing of 16S rRNA regions. The detection of metabolites from fecal samples was performed using high-performance liquid phase chromatography and gas chromatography coupled with tandem mass spectrometry. Intestinal microbiota community and metabolite analysis both showed separation of cirrhotic patients from control participants, moreover, the microbiota–metabolite correlations changed in cirrhotic patients. Fecal microbiota from cirrhotic patients, with the reduced diversity, contained a decreased abundance of Bacteroidetes and an increased abundance of Firmicutes and Proteobacteria compared with the normal samples. Analysis of metabolome revealed a remarkable change in the metabolic potential of the microbiota in cirrhotic patients, with specific higher concentrations of amine, unsaturated fatty acid, and short-chain fatty acids, and lower concentrations of sugar alcohol and amino acid, suggesting the initial equilibrium of gut microbiota community and co-metabolism with the host were perturbed by cirrhosis. Our study illustrated the relationship between fecal microbiota composition and metabolome in cirrhotic patients, which may improve the clinical prognosis of cirrhosis.

## Introduction

The liver plays a pivotal role in material metabolism, such as synthesis of proteins, detoxification, and storage. Liver lesions will inevitably lead to a change in related metabolic network. Cirrhosis, often preceded by hepatitis and steatosis, is a condition in which the liver does not function properly due to long-term damage. Cirrhosis resulted in 1.2 million deaths in 2013, up from 0.8 million deaths in 1990 (GBD 2013 Mortality and Causes of Death Collaborators, 2015). Independent of the cause, treatment of cirrhosis partly depends on the underlying cause, with the principle of preventing worsening and complications.

The liver anatomy exhibits its close interaction with the gut (Szabo, 2015). Liver dysfunction causes serious complications, such as bacteremia, ascites, and hepatic encephalopathy, accompanied by increased intestinal permeability and changed intestinal microenvironment, resulting in intestinal microbiota dysbiosis and dysfunction (Garcovich et al., 2012; Quigley et al., 2013; Minemura and Shimizu, 2015). Human intestinal microbiota was an important organ which has been neglected for a long time. The human gut harbors up to 100 trillion microbes and encode 100-fold more unique genes than the human genome, which were regarded as the “second genome” of the body (Martínez et al., 2013). Microbial communities were dominated by members of just two bacterial phyla, Bacteroidetes and Firmicutes (Eckburg et al., 2005). Obligate anaerobes, such as *Bacteroides*, *Bifidobacterium*, *Bacillus*, and *Peptostreptococcus*, account for 99.9% of the intestinal microbiota, with *Bacteroides* and *Bifidobacterium* accounting for 55% of them. Most of the resident microbes in the gut have a profound influence on human physiology and nutrition. The gut flora function as a bioreactor for autonomous and co-metabolism and can regulate responses within the host to external stimuli, and perform useful functions (Del Chierico et al., 2014), such as fermenting undigestible compounds to produce energy, training the immune system, providing nutrition for intestinal epithelial cells, preventing growth of pathogens, providing vitamins (such as biotin and vitamin K) for host. There were accumulating evidence available implicating that extensive modification and imbalances of the gut microbiota and its microbiome were responsible for many human disease, such as bacterial endocarditis, heart disease, cancer, pneumonia, atherosclerosis, preterm low birth weight, chronic kidney disease, obesity, and pancreatic cancer (Steinhoff, 2005).

In the past few years, more and more research were focused on the intestinal microbiota community in patients with hepatopathy. Ahluwalia et al. (2016) enrolled 40 controls and 147 cirrhotic patients (87 with hepatic encephalopathy), and researched on the relationship between stool microbiota and brain magnetic resonance imaging (MRI), with the results shown negative linkages between autochthonous families and positive ones between Enterobacteriaceae to MR spectroscopy and hyperammonemia-associated astrocytic changes. Grąt et al. (2016) analyzed the gut microbial profile from 15 patients with hepatocellular cancer (HCC) and 15 non-HCC patients, which suggested that the overgrowth of *E coli* in the gut may contribute to the process of hepatocarcinogenesis. Usami et al. (2013) collected fecal samples from 46 hepatic cancer patients, followed by the evaluation of fecal constituents and lipid and fatty acid concentrations, and suggested that intestinal microbiota affected serum fatty acid metabolism and were modified by liver disorders. Bajaj et al. (2015) collected fecal and salivary samples from 102 cirrhotic patients (43 previous hepatic encephalopathy) and 32 controls, followed by stool/saliva microbiome composition and function analysis, as well as systemic inflammatory evaluation, to evaluate the oral microbiome in cirrhosis and compare with stool microbiome. Fecal samples were collected from 15 compensated cirrhotic patients and 15 age-matched controls to evaluate changes in stool microbiota composition and function in cirrhotic patients after omeprazole therapy, and they found that omeprazole was associated with a microbiota shift and functional change in the distal gut in compensated cirrhotic patients that could set the stage for bacterial overgrowth (Bajaj et al., 2014). Chen et al. (2014) researched on fecal samples from 12 alcoholic cirrhosis patients, 18 hepatitis B virus related cirrhosis patients, and 12 normal controls using a specific functional gene array, which suggested that the functional composition of fecal microbiota was heavily influenced by cirrhosis, especially by alcoholic cirrhosis. Grąt et al. (2014) confirmed the enrichment of *lactobacilli* in the gut microflora of liver transplant candidates with cirrhosis as revealed by intestinal flora component analysis. Seventeen cirrhotic patients and 24 healthy individuals were recruited and subjected to fecal metabolites detection, which suggested the accompany of malabsorption with disorders of fatty acid metabolism, potentially due to changes in gut microflora (Huang et al., 2013). Park et al. (2016) suggested that alcoholic liver disease should be considered in the aspect of both gut health and chronic systemic low-grade inflammation. Given the above, hepatopathy had a significant impact on the component and function of gut microbiota via gut–liver axis. However, few intensive studies have investigated the Child-Turcotte-Pugh (CTP) score related metabolic interactions between the gut and liver, as well as the correlation between gut flora component and metabolism. Our study highlighted the association and communication between gut flora community and metabolism and researched how the intestinal microbiota regulated systemic metabolic changes in cirrhotic patients.

## Materials and Methods

### Study Participants and Fecal Samples

The severity and prognosis of cirrhosis was classified with the CTP scoring system, which was based on bilirubin, albumin, prothrombin time, presence and severity of ascites, and encephalopathy. In this study, a total of 13 individuals, including 10 cirrhotic patients (five of CTP score A, five of CTP score B) and three healthy individuals were enrolled with informed consent. Class A patients had a favorable prognosis, while symptome of class B patients was more severe clinically. All the participants were 40–60 years old, with a normal body mass index and had no dietary preferences. Cirrhosis was diagnosed by liver biopsy in all patients. None of these patients had comorbid diseases. All healthy individuals had normal liver biochemistry tests without evidence of hepatic or other diseases. Exclusion criteria included a history of antibiotics, probiotics, steroids, or other hormones ingestion (including oral, intramuscular or intravenous) within the previous 3 months of stool sampling. This study was approved by the Institutional Review Board of Affiliated Hospital of Academy of Military Medical Sciences. All participants signed an informed consent form prior to entering the study. The study conformed to the ethical guidelines of the 1975 Declaration of Helsinki.

### 16S rRNA Gene Amplicon Library Preparation and Sequencing

Microbial DNA extraction was performed using the QIAamp DNA Stool Mini Kit (Cat No: 51504) with 220 mg of fecal sample. Metagenomic sequencing of 16S rRNA gene V1–V3 region was performed with the MiSeq platform (Illumina Inc., San Diego, CA, USA) using version 3 (300 × 2) chemistry on the MiSeq instrument (Illumina) according to manufacturer’s instructions (Kozich et al., 2013). Library preparation and sequencing were performed as previously described (Mwaikono et al., 2016).

#### Metabolite Extraction and Separation

Fresh fecal samples were taken from all participants for metabolites extraction. Metabolites extraction was performed by adding 1.2 ml of cold (-80°C) high performance liquid chromatography (HPLC)-grade methanol to fecal samples. Samples were vortex-mixed followed by sonicated for 30 s (Sonicator^®^ 3000; Misonix) on liquid nitrogen. This protocol was repeated five times with a 60-min storage period at -80°C between each cycle. The final pellet was removed by centrifugation at 16,000 rpm for 10 min at 4°C, and the supernatant was stored at -80°C. The methanolic extract was centrifuged at 13,000 rpm for 20 min at 4°C to precipitate any solid impurity. The supernatant was analyzed by high-performance liquid phase chromatography and gas chromatography coupled with tandem mass spectrometry (HPLC-GC/MS-MS, Metabolon, Inc., Durham, NC, USA) as described previously (Sha et al., 2010).

#### Metabolites Identification and Data Processing

Samples were analyzed in one randomized run, during which time they were kept in the liquid phase chromatography autosampler at 4°C. Background noise and unrelated ions were removed from the resulting data file using the Molecular Feature Extraction (MFE) tool in the Mass Hunter Qualitative Analysis software (B.06.00, Agilent). Filtering and alignment of primary data were performed with Mass Profiler Professional software (version 13.0, Agilent). The multivariate analyses were performed using SIMCA-P+ software (12.0.1.0, Umetrics) to generate a partial least squares discriminant analysis (PLS-DA) model with all the variables, and quality controls (QCs) were predicted into this model. These data were then represented in a hierarchical condition tree (HCT). Their identities were confirmed by comparing the fragments that were obtained with the structure of the proposed compound in the MS/MS spectra in a public database (METLIN: https://metlin.scripps.edu/metabolites_list.php) or against commercially available standards. Wilcoxon rank-sum test method was used to assess the metabolites with significant differences in abundance between patients and the normal.

## Results

### Methodology and Study Population

A compositional survey of metabolites from fecal samples was used to identify cirrhosis related characteristics in microbes’ metabolic activity. In this study, we recruited and investigated cirrhotic patients with CTP score of A (AP, *n* = 5) and CTP score of B (BP, *n* = 5), and normal individuals (NM, *n* = 3). The information and characteristics of all the recruited subjects were reported in **Table 1**. A full description of the methods is available in the Section “Materials and Methods.”

**Table 1:** Characteristics of the study population.

	NM			AP					BP				
	NM1	NM2	NM3	AP1	AP2	AP3	AP4	AP5	BP1	BP2	BP3	BP4	BP5
Sampling time	5/1/2016	7/1/2016	5/1/2016	5/1/2016	5/1/2016	7/1/2016	7/1/2016	7/1/2016	7/1/2016	7/1/2016	7/1/2016	7/1/2016	5/1/2016
Age (years)	43	51	46	52	54	57	44	49	50	51	46	47	53
Gender	Female	Male	Female	Male	Male	Male	Male	Female	Female	Male	Male	Male	Male
Body mass index (kg/m^2^)	24.2	19.6	19.1	24.2	23.3	24.7	23.6	20.9	22.5	23.3	20.9	19.6	23.9
Ascites	–	–	–	2	2	1	1	1	2	1	3	2	2
Prothrombin time (second)	–	–	–	13.1	12.9	12.9	13.5	10.3	11.6	12.4	12.1	14.5	12.2
Prothrombin time score	–	–	–	1	1	1	1	1	1	1	1	1	1
Albumin (g/L)	–	–	–	31	39	31	35	39	31	42	31	31	40
Albumin score	–	–	–	2	1	1	1	1	1	1	3	2	1
Tbil (μmol/L)	–	–	–	20.3	19.7	21.6	29.1	18.2	17	13.4	10.3	11.2	12.9
Tbil score	–	–	–	1	1	1	1	1	1	3	1	1	1
Child-Turcotte-Pugh score	–	–	–	6	5	5	5	5	8	8	9	7	7

### Cirrhosis Related Composition of the Intestinal Microbiota

**FIGURE 1:**
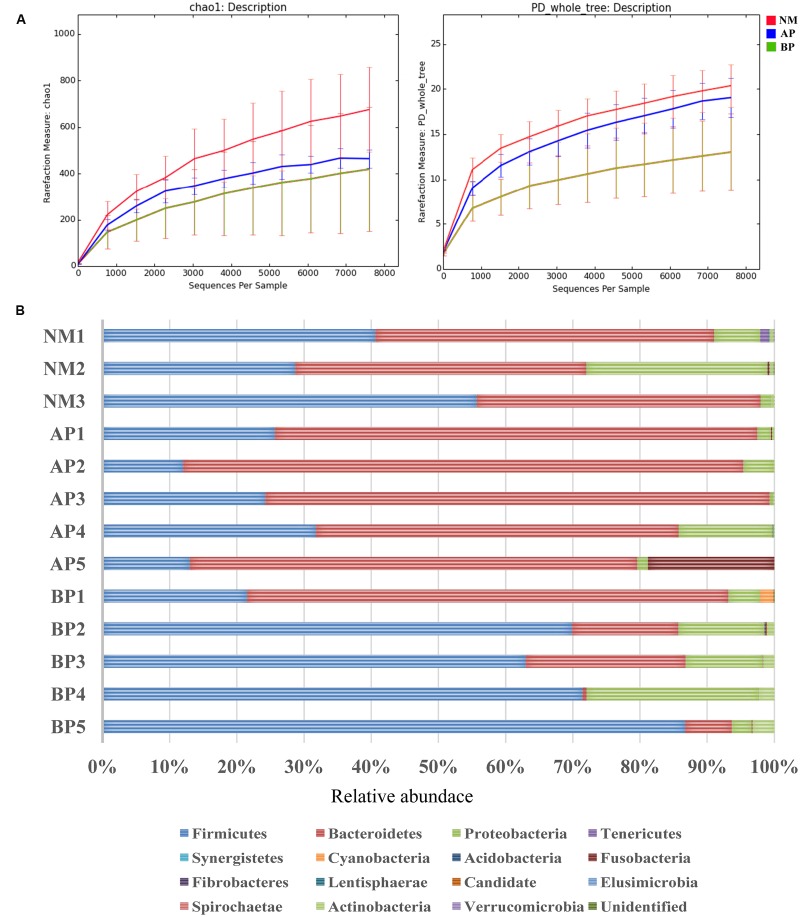
**Characteristics of fecal microbiota community from the study population. (A)** Averaged number of richness (Chao 1) and diversity (PD, whole tree) of fecal samples from the subjects. The centered line represents the median value of NM (red), AP (blue), and BP (green), the top and bottom represent the 95th and 5th percentile of the data, respectively. **(B)** Relative abundance (%) of predicted bacterial phylum in the fecal microbiota community from cirrhotic patients and the normal.

The microbial composition of fecal samples was analyzed by pyrosequencing of 16S rRNA gene. The bacterial community Chao 1 diversity and phylogenetic diversity measures were determined, which revealed a significant decrease in microbial diversity from cirrhotic patients, even more remarkable in BP patients (**Figure 1A**). Fecal microbiota community, in especial Firmicutes, Proteobacteria, and Bacteroidetes, from cirrhotic patients was clearly different from the normal (**Figure 1B**). Samples from BP patients contained a significant reduction of Bacteroidetes and increase in Firmicutes and Proteobacteria compared with the normal. Overall, fecal microbiota component from AP patients had no significant difference compared with the normal, which may largely due to that gut microbiota were in compensatory state at the early stage of disease. In the family level, fecal microbiota from the cirrhotic patients contained a remarkable absence of Bacteroidaceae, and enhancement of Enterobacteriaceae, Veillonellaceae, and Streptococcaceae.

### Cirrhosis Related Changes in the Predicted Metabolites

**FIGURE 2:**
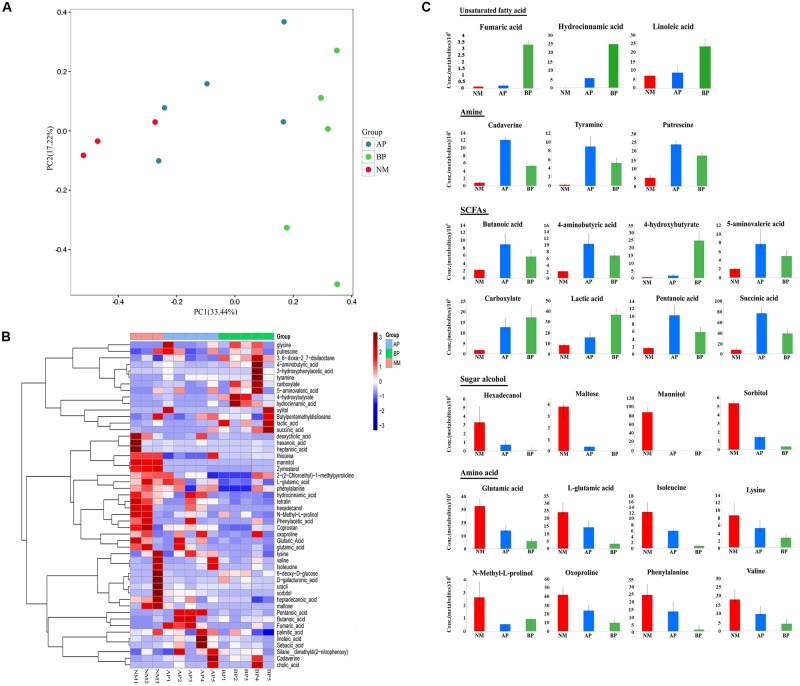
**Characteristics of metabolites from fecal samples of cirrhotic patients compared with the normal. (A)** Principle component analysis (PCA). The analysis was based on unweighted UniFrac distance matrix of all metabolites concentrations from fecal samples of the cirrhotic patients (AP and BP) and the normal. **(B)** Color-coded heat map displaying the cirrhosis related relative fecal metabolites amounts compared to the normal. Hierarchical clustering of the metabolic profiling data at NM (red bars), AP (blue bars), BP (green bars). The color scale represents the scaled abundance of each metabolite, denoted as *Z*-score, with red and blue indicated increased and decreased concentrations, respectively. **(C)** Cirrhosis related common sets of metabolic perturbations. Fecal samples of the subjects were taken for metabolic profiling. The values displayed are the average concentrations of each metabolite. Columns are colored according to the groups: red: NM; blue: AP; green: BP. Data are shown as mean values ± SD for each group.

We profiled the fecal microbiota metabolome of the subjects to explore global metabolic alterations associated with liver cirrhosis. A total of 108 metabolites were robustly identified, and 51 metabolites had different abundance between cirrhotic patients and the normal (**Supplementary Table S1**). The fecal samples of cirrhotic patients were clearly differentiated from the normal as shown by the principal coordinates analysis based on unweighted unifrac distances (**Figure 2A**). To further elucidate the CTP-dependent impact on the fecal microbiota metabolism, we separately performed hierarchical clustering on the relative metabolite concentration for the detected metabolites which have suggested that fecal samples from the normal could be clearly distinguished from the samples taken from the patients (**Figure 2B**). The fecal samples from patients were characterized by a higher abundance of amino acids metabolism. A significant increase in the amount of glycine was detected in patients, whereas other amino acids, such as glutamic acid, L-glutamic acid, isoleucine, lysine, *N*-methyl-L-prolinol, oxoproline, phenylalanine, and valine, were detected in significantly decreased amounts. In addition, enhanced carbohydrate metabolism, especially the catabolism of some sugar alcohol, such as hexadecanol, maltose, mannitol, sorbitol, was observed in cirrhotic patients, and correspondingly, its metabolites, such as organic acid, were detected with a higher abundance.

In addition, we explored the alterations associated with cirrhosis (**Figure 2C**). The relative concentrations of some unsaturated fatty acids (fumaric acid, hydrocinnamic acid, and linoleic acid) were generally seen to increase in the fecal metabolites from cirrhotic patients, especially in class B patients. Cirrhosis-specific trends were more evident for amine (cadaverine, tyramine, putrescine), with the cirrhotic samples showing a large number of increase compared to the normal. An increase in concentration of some short-chain fatty acids (SCFAs) and their compounds (butanoic acid, 4-aminobutyric acid, 4-hydroxybutyrate, 5-aminovaleric acid, carboxylate, lactic acid, pentanoic acid, succinic acid), as well as bile acids were evident in the metabolites from patients. In contrast, the median values for some amino acids, such as glutamic acid, L-glutamic acid, isoleucine, lysine, *N*-methyl-L-prolinol, oxoproline, phenylalanine, valine, were generally found to decrease in the fecal samples from cirrhotic patients. In addition, we detected some sugar alcohol (hexadecanol, maltose, mannitol, sorbitol) were significantly decreased in cirrhotic patients.

### Cirrhosis Related the Microbiota–Metabolite Correlations

**FIGURE 3:**
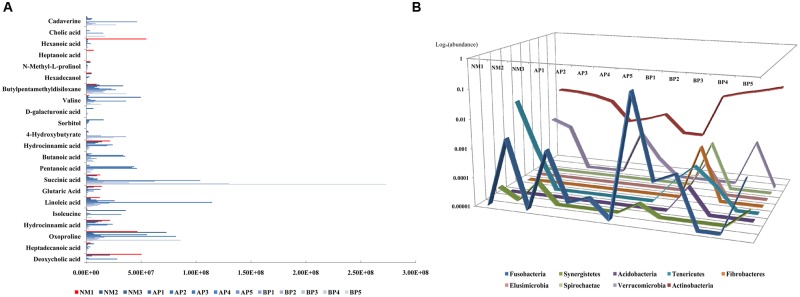
**The correlation analysis between the abundance of fecal metabolites (A)** and fecal microbiota composition, with the data shown as logarithm value, **(B)** among the subjects.

Correlation analysis was conducted to further explore the association between fecal microbiota and metabolites. Overall, the microbiota–metabolite correlations changed in cirrhotic patients (**Figure 3**), indicating that the initial equilibrium of co-occurring microbial species was perturbed by cirrhosis. We found that Fusobacteria, enhanced in the fecal microbiota community in cirrhotic patients, was positively correlated with cadaverine (*R* = 0.85) and cholic acid (*R* = 0.6). Tenericutes, contained a decreased abundance in cirrhotic patients, was positively correlated with hexanoic acid (*R* > 0.99), heptanoic acid (*R* > 0.99), *N*-methyl-L-prolinol (*R* = 0.62), and hexadecanol (*R* = 0.62). Synergistetes, had a decreased abundance in cirrhotic patients, was positively correlated with butylpentamethyldisiloxane (*R* = 0.77), valine (*R* = 0.67), D-galacturonic acid (*R* = 0.63), sorbitol (*R* = 0.61). Acidobacteria, Fibrobacteres, Elusimicrobia, and Spirochaetae were all positively correlated with 4-hydroxybutyrate (*R* = 0.93) and hydrocinnamic acid (*R* = 0.93). In addition, butanoic acid and pentanoic acid were negatively correlated with almost all the bacteria, except Bacteroidetes (*R* = 0.4). Succinic acid was positively correlated with Actinobacteria (*R* = 0.8). Glutaric acid was positively correlated with Synergistetes (*R* = 0.67) and Tenericutes (*R* = 0.64). Linoleic acid was positively correlated with Lentisphaerae (*R* = 0.6), which is a phylum of bacteria closely related to Verrucomicrobia. Isoleucine was positively correlated with Synergistetes (*R* = 0.67) and Fusobacteria (*R* = 0.62). Hydrocinnamic acid was positively correlated with Synergistetes (*R* = 0.67) and Verrucomicrobia (*R* = 0.61). Oxoproline was positively correlated with Proteobacteria (*R* = 0.79) and Synergistetes (*R* = 0.67). Heptadecanoic acid was positively correlated with Verrucomicrobia (*R* = 0.6). Deoxycholic acid was positively correlated with Tenericutes (*R* = 0.82), Verrucomicrobia (*R* = 0.62).

## Discussion

The host and its gut microbiota coproduce a wide spectrum of small molecules during the cometabolism of compounds in diet and xenobiotics, and the intestinal cometabolism play critical roles in communication and interaction between host organs and the host’s gut flora symbionts. Our data indicated pronounced shifts in gut bacterial community and metabolome associated with cirrhosis. The metabolic materials from cirrhotic fecal samples had a higher abundance of amine, unsaturated fatty acid, and SCFAs, whereas a lower abundance of sugar alcohol and amino acid, similar with previous research from Wei et al. (2013), which suggested that fecal microbiota from cirrhotic patients contained an enrichment in amino acid transport and metabolism, energy production and conversion, secondary metabolites biosynthesis, transport and catabolism as revealed by metagenomic approach.

Some amino acids were significantly decreased in fecal metabolites from cirrhotic patients. Glutamic acid is non-essential for human, meaning the body can synthesize it, and 95% of the dietary glutamate is metabolized by intestinal cells in a first pass. Isoleucine, lysine, phenylalanine, and valine are essential amino acids for human because they cannot be created from other compounds by the human body and so must be taken in as food. Amine was the main products of decomposition of amino acid. Cadaverine, similar with putrescine, is the decarboxylation product of the amino acid lysine and is a foul-smelling toxic diamine compound produced by the putrefaction of animal tissue. Tyramine is a naturally occurring monoamine compound and trace amine derived from the amino acid tyrosine. Production of cadaverine, tyramine, putrescine in human gut was strongly associated with intestinal microbiota which can decompose amino acids, including Fusobacteria, *Proteus, Bacillus*, *Pseudomonas*, and so on, and those bacteria can form protease and peptidase only when a large number of breeding. It has been recognized that Fusobacteria is involved in various human infections causing tissue necrosis and septicemia (Parte, 2014). In our study, cadaverine, showing a large number of increase compared to the normal in the cirrhotic samples, was positively correlated with Fusobacteria, and the amino acids were significantly decreased in cirrhotic fecal metabolites, indicating an enhanced amino acids-degrading activity of Fusobacteria in human gut from cirrhotic patients.

Hexadecanol, positively correlated with Tenericutes, is a fatty alcohol with the formula CH_3_(CH_2_)_15_OH. Maltose is a disaccharide formed from two units of glucose. In human gut, maltose is broken down by the enzyme maltase so that there are two glucose molecules from which the glucose metabolism obtains energy. Mannitol, as one of the most abundant energy and carbon storage molecules in nature, was produced by plethora of organisms, including bacteria, fungi, algae, and many plants (Song and Vieille, 2009). Sorbitol is a sugar alcohol with a sweet taste, which could be metabolized slowly by human body. Sorbitol and valine were both positively correlated with Synergistetes, which suggested to be opportunistic pathogens and inhabit a majority of anaerobic environments, including gastrointestinal tracts, have also been implicated in gastrointestinal infections and soft tissue infections (Marchandin et al., 2010).

SCFAs are end-product of anaerobic fermentation, called saccharolytic fermentation, performed by beneficial colonic bacteria that feed on, or ferment prebiotics, which are plant products that contain adequate amounts of dietary fiber (Van Immerseel et al., 2005). SCFAs are significant sources of energy for gut enterocytes, providing a major source of useful energy and nutrients for human, helping the body to absorb essential dietary minerals such as calcium, magnesium, and iron (Clarke et al., 2014), influencing the gastrointestinal barrier function to reduce pathogenic bacterial colonization (Brahe et al., 2013), benefiting the colonocytes by increasing energy production and cell proliferation, and may protect against colon cancer (Charalampopoulos et al., 2002). Moreover, emerging research suggested the role for SCFAs in reducing and controlling inflammation (Samuel et al., 2008). Miyoshi et al. (2011) confirmed that oral tributyrin administration increased plasma butyrate level in the portal vein by one to two orders of magnitude, and attenuated NF-κB activation and lipopolysaccharide (LPS)-induced acute liver tissue injury in rats. Two reports from Bloemen et al. (2009, 2010) confirmed the role of the gut and liver in SCFAs exchange by measuring portal and hepatic venous SCFA concentrations *in vivo*, which were shown that intestinal SCFAs release by the gut was equally to hepatic uptake, liver of cirrhosis was able to use butyrate and propionate, most likely preventing increased systemic concentrations. In this study, we found that butanoic acid and pentanoic acid were negatively correlated with almost all the bacteria, except Bacteroidetes. Opportunistic pathogens were overexpressed in the fecal microbiota community from cirrhotic patients, still there were Bacteroidetes which metabolize carbohydrate into SCFAs to benefit the intestine, playing a compensatory role for the dysfunction of intestinal microenvironment. 4-Hydroxybutyrate was positively correlated with Acidobacteria, Fibrobacteres, Elusimicrobia, and Spirochaetae. Cells produce 4-hydroxybutyrate by reduction of succinic semialdehyde via succinic semialdehyde reductase, and 4-hydroxybutyrate could be degraded into succinic acid under the action of alcohol dehydrogenase (ADH) and acetaldehyde dehydrogenase (ALDH). Succinic acid was positively correlated with Actinobacteria, and followed by Firmicutes. Actinobacteria, which had an increased abundance in the fecal microbiota from cirrhotic patients, are of great economic importance to humans and are recognized as the producers of many bioactive metabolites useful to humans. In addition, Hexanoic acid and heptanoic acid were both positively correlated with Tenericutes. Glutaric acid was the metabolite production in both fatty acid degradation (map00071) and lysine degradation (map00310) pathway and positively correlated with Synergistetes and Tenericutes. Increased abundance of SCFAs in fecal metabolites suggested the enhanced ability of carbohydrate metabolism in cirrhotic patients. Due to the complex component of gut microbiota, various SCFAs were detected positively correlated with different bacterial taxa. Our results showed that fermentative subsets of gut bacterial taxa might be selectively stimulated by carbohydrate and might therefore be capable of consuming plant-derived saccharides in human gut.

Bile acids, composed of individual bile acid moieties, mucous, phospholipids, and biliverdin (Begley et al., 2005), function as systemic signaling molecules, and their main physiological roles in the small intestine are emulsification of fats, release of fat-soluble vitamins, regulation of cholesterol metabolism (Swann et al., 2011; Vaquero et al., 2013). Interdependent relationship existed between intestinal microbiota and bile acids. On the one hand, intestinal microbiota was vital for the transformation of bile acids. Conjugated bile salts secreted into the enteric cavity are hydrolyzed and dehydroxylated by bile salt hydrolases (BSHs), predominantly secreted by *Bacteroides* and Fusobacteria (Hofmann, 1999), and as a result, cholic acids were converted into deoxycholic acid, and chenodeoxycholic acid into lithocholic acid. On the other hand, the dissociation of conjugated bile acids can provide carbon source, nitrogen source, and energy for intestinal microbiota. The amount of fecal bile acids was influenced by many factors. In cirrhotic patients, abated liver function and the dysbiosis and dysfunction in fecal microbiota resulted in the declined role of dissociation and dehydroxylation for bile acid. Bile acid metabolism was disordered, and the reabsorption in the colon was decreased, so more bile acid was detected in the fecal metabolites.

Above all, it should be noticed that albeit the gut flora function is always changing, it is also relatively stable, and has evolved specialized core functions that are maintained under different conditions (Moya and Ferrer, 2016). Cirrhotic patients have weak constitution, and as a result the body tissues need to enhance their expenditure and degradation of materials to partially compensate for the increased demand for nutrients and energy, and the fecal microbiota seemed to play compensatory and regulation roles, which may provide more nutrients and energy resources for the patients. As a result, most amino acid and sugar alcohol, which were few detected from cirrhotic samples, were further metabolized into a large number of small molecule metabolites, such as amine, unsaturated fatty acid, and SCFAs.

## Conclusion

Altogether, our data indicated that the relationship between some gut flora and humans is not merely non-harmful coexistence, but rather a symbiotic relationship (Del Chierico et al., 2014). The liver is the central organ in host metabolism. At present, treatment of cirrhosis is partly based on the principle of preventing worsening and complications depending on the underlying cause. Our study will improve the prognosis and provide fundamental basis for interventions of cirrhosis progress and related infectious and non-infectious complications, including metabolic disorders induced by the gut–liver axis, especially alcoholic liver disease (ALD), non-alcoholic fatty liver disease (NAFLD) and hepatocarcinogenesis.

## Author Contributions

YS and XW designed research; XW, HL, WL, and SJ performed research; XW, BL, JY, and XZ contributed new reagents or analytic tools; XW and JL analyzed data; XW and YS wrote the paper.

## Conflict of Interest Statement

The authors declare that the research was conducted in the absence of any commercial or financial relationships that could be construed as a potential conflict of interest.

## Supplementary Material

TABLE S1 **Characteristics of the 51 metabolites identified differentially between cirrhotic patients and the normal**.

## Abbreviations

ADH: alcohol dehydrogenase
ALD: alcoholic liver disease
ALDH: acetaldehyde dehydrogenase
CTP: Child-Turcotte-Pugh
EPA: eicosapntemacnioc acid
HCC: hepatocellular cancer
HCT: hierarchical condition tree
HPLC-GC/MS-MS: high-performance liquid phase chromatography and gas chromatography coupled with tandem mass spectrometry
LPS: lipopolysaccharide
MFE: molecular feature extraction
NAFLD: non-alcoholic fatty liver disease
PCA: principle component analysis
PLS-DA: partial least squares discriminant analysis
QC: quality control
SCFAs: short-chain fatty acids
